# Pulsed Ultrasound Differentially Stimulates Somatosensory Circuits in Humans as Indicated by EEG and fMRI

**DOI:** 10.1371/journal.pone.0051177

**Published:** 2012-12-04

**Authors:** Wynn Legon, Abby Rowlands, Alexander Opitz, Tomokazu F. Sato, William J. Tyler

**Affiliations:** Virginia Tech Carilion Research Institute and School of Biomedical Engineering and Sciences, Virginia Tech, Roanoke, Virginia, United States of America; University of Montreal, Canada

## Abstract

Peripheral somatosensory circuits are known to respond to diverse stimulus modalities. The energy modalities capable of eliciting somatosensory responses traditionally belong to mechanical, thermal, electromagnetic, and photonic domains. Ultrasound (US) applied to the periphery has also been reported to evoke diverse somatosensations. These observations however have been based primarily on subjective reports and lack neurophysiological descriptions. To investigate the effects of peripherally applied US on human somatosensory brain circuit activity we recorded evoked potentials using electroencephalography and conducted functional magnetic resonance imaging of blood oxygen level-dependent (BOLD) responses to fingertip stimulation with pulsed US. We found a pulsed US waveform designed to elicit a mild vibration sensation reliably triggered evoked potentials having distinct waveform morphologies including a large double-peaked vertex potential. Fingertip stimulation with this pulsed US waveform also led to the appearance of BOLD signals in brain regions responsible for somatosensory discrimination including the primary somatosensory cortex and parietal operculum, as well as brain regions involved in hierarchical somatosensory processing, such as the insula, anterior middle cingulate cortex, and supramarginal gyrus. By changing the energy profile of the pulsed US stimulus waveform we observed pulsed US can differentially activate somatosensory circuits and alter subjective reports that are concomitant with changes in evoked potential morphology and BOLD response patterns. Based on these observations we conclude pulsed US can functionally stimulate different somatosensory fibers and receptors, which may permit new approaches to the study and diagnosis of peripheral nerve injury, dysfunction, and disease.

## Introduction

Responses of the human somatosensory system to a variety of stimuli have been studied extensively in research and are useful in clinical sensory testing [1]. Mechanical stimulation of somatosensory circuits can be achieved through simple punctate stimulation, the application of textured surfaces to the skin, and vibration. These stimuli activate different mechanoreceptors of the skin and underlying tissues [2], [3], [4]. Thermoreceptors, nociceptors, and polymodal receptors can be stimulated using peltier chips, heat lamps, and lasers. The brain responds to these various stimulus modalities by exhibiting somatosensory-evoked potentials (SEPs), contact heat-evoked potentials (CHEPs) and laser-evoked potentials (LEPs) depending on the stimulation approach used [1], [5], [6], [7]. Collectively these evoked potentials (EPs) can exhibit unique spatial and temporal waveform morphologies arising from the different receptor populations and fiber tracts activated. For example, mechanical stimuli have been demonstrated to preferentially activate primary and secondary somatosensory cortices while thermal and noxious stimulation often activates additional areas including the cingulate and insular cortices [5], [8], [9], [10].

Interestingly, ultrasound (US) has been reported to stimulate a wide variety of subjective somatosensations in humans [11], [12], [13], as well as EPs in response to painful ultrasonic stimuli [14], [15]. Based on those observations and our previous ones that US can directly stimulate central neurons [16], it has been hypothesized the spatiotemporal energy profile of US waveforms may enable the targeted stimulation of specific protein ion channels and receptors [11], [12], [17], [18]. Based on that hypothesis, US applied to the periphery should be capable of differentially eliciting ultrasound-evoked potentials (USEPs) and BOLD responses, which share characteristics similar to conventional somatosensory stimulus modalities. Thus, in the present study we focused on characterizing brain activity patterns elicited in response to stimulation of peripheral somatosensory circuits using different US waveforms while acquiring EEG recordings and fMRI BOLD imaging sequences. Our data illustrate the manipulation of US waveform parameters can induce diverse sensations concomitant with functional changes in brain activity patterns as indicated by differences in USEPs and fMRI BOLD responses.

## Materials and Methods

### Ethics Statement

All procedures were approved by the Institutional Review Board at Virginia Tech.

### Subjects

Twenty subjects (13 male, 7 female, aged 21–59, mean age 32.6±10.4) provided written informed consent to participate in the study. A subset of these subjects underwent EEG recordings (N = 5) and fMRI scanning (N = 5) during fingertip stimulation with US. None of the subjects reported any history of neurological or musculoskeletal impairments and all were right-hand dominant [19].

### Peripheral ultrasonic neurostimulation

**Figure 1:**
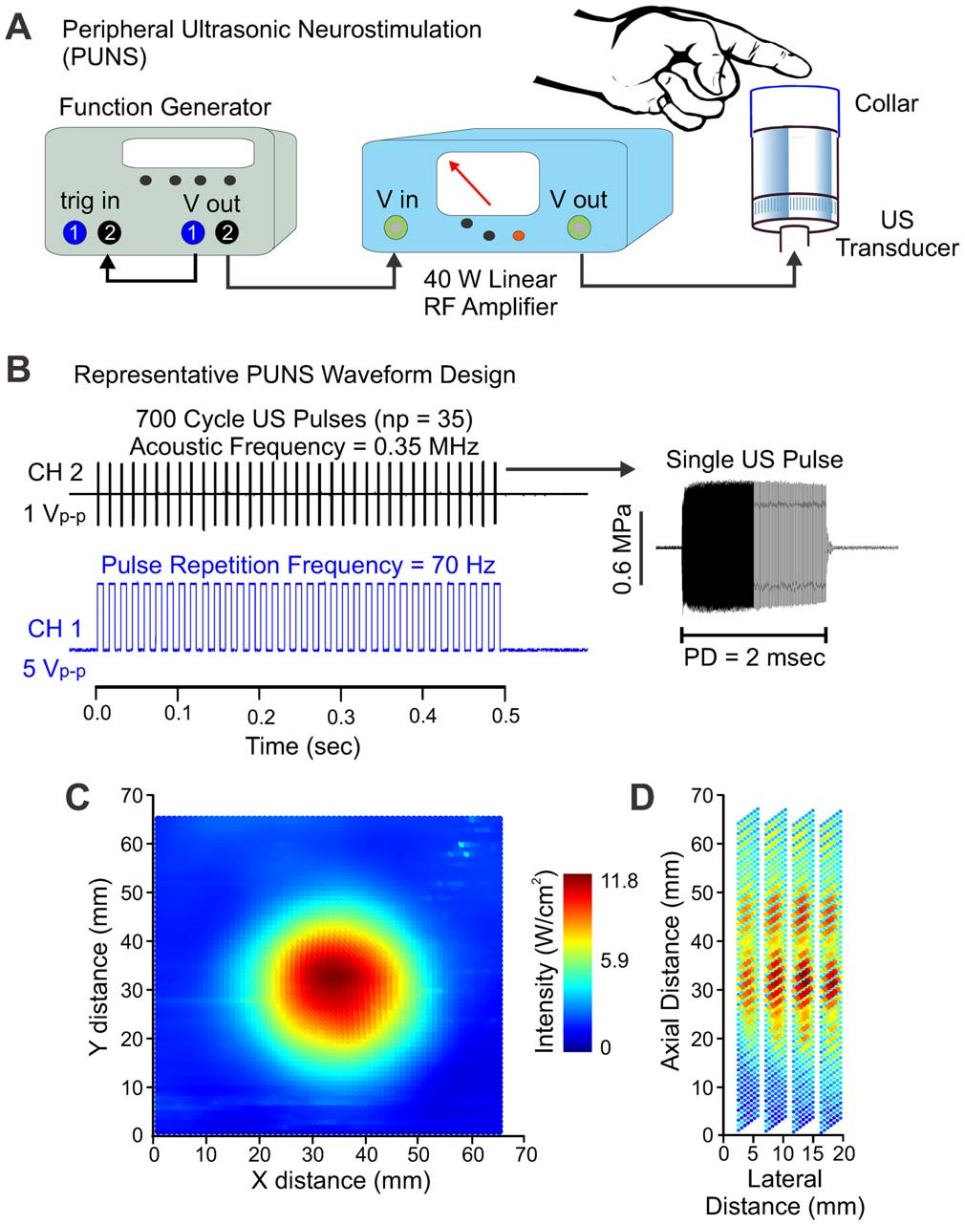
Design and delivery of pulsed ultrasound waveforms for somatosensory stimulation. (**A**) Example of experimental setup implemented to achieve peripheral ultrasonic stimulation (PUNS). Note the illustrations are not drawn to scale. (**B**) Illustration of the waveform strategy used to generate the PUNS-M stimulus, which elicited a brief mechanical buzzing sensation. The *top-black* trace illustrates a one volt peak-to-peak (V_p-p_) square-wave signal originating from channel two (CH 2) of the function generator to drive acoustic pulses. Each pulse contained 700 cycles occurring at a frequency of 0.35 MHz. This voltage waveform was fed into the input of the RF amplifier as shown in **A** above. A single ultrasound pulse emitted from the transducer is shown at *right* (MPa  =  megapascals; PD  =  pulse duration). The *bottom-blue* trace illustrates 35, five V_p–p_ square-waves originating from channel one (CH 1) of the function generator. The signal generated by CH1 served as the pulse repetition frequency waveform and was used to trigger each CH2 pulse. (**C**) An intensity plot showing the lateral (XY) acoustic output profile from the ultrasound transducer during a PUNS-M waveform is shown using a pseudo-color look-up table. (**D**) Similar to **C**, a pseudo-color intensity plot illustrates the acoustic power delivered from the ultrasound transducer during a PUNS-M waveform as a function of lateral distance across the face of the transducer, as well as axial distance from the face of the transducer.

Peripheral ultrasonic neurostimulation (PUNS) waveforms were generated using a two-channel, 2 MHz function generator (BK Precision Instruments) similar to previously described methods [20]. Briefly, channel one was designated as the pulse repetition frequency (PRF) component of the waveform and channel two was used to generate the acoustic frequency (A*_f_*) of the stimulus waveform with channel one serving as the trigger input driving channel two. The pulse duration (PD) of PUNS waveforms were set by adjusting the number of cycles per pulse on channel two while the stimulus duration was set by adjusting the number of pulses (np) on channel one. The output from channel two was sent through a 40 W linear RF amplifier (E&I 240L; Electronics & Innovation) before being sent to a custom 0.35 MHz MR-compatible gas matrix piezocomposite ultrasound transducer (Ultran Group, State College, Pennsylvania USA). Transducers were affixed with a polypropylene collar to create a reservoir extending 30 mm above the face of the transducer. The reservoir was filled with acoustic coupling gel and subjects were instructed to place his/her finger on the surface of the gel (Figure 1A).

We synthesized two distinct PUNS waveforms where one waveform was intended to evoke a slight mechanical sensation (designated as PUNS-M) and the other waveform was designed to elicit a thermal sensation; designated as PUNS-T. We measured the acoustic intensity profiles of PUNS waveforms using a calibrated hydrophone (HNR-0500, Onda Corporation, Sunnyvale, CA) as previously described [17], [20]. The PUNS-M waveform had the following parameters: A*_f_*  = 0.35 MHz, PD  = 2 msec, PRF  = 70 Hz, and np  = 35 to produce a stimulus duration of 0.5 sec yielding a spatial-peak temporal-average intensity (I_SPTA_) of 11.8 W/cm^2^ (Figure 1B–D). The PUNS-T waveform had the following parameters: A*_f_*  = 0.35 MHz, PD  = 10 msec, PRF  = 100 Hz, and np  = 100 to produce a stimulus duration of 1.0 sec yielding an I_SPTA_ of 54.8 W/cm^2^.

Participants rated the sensations experienced in response to ultrasonic stimulation using two separate visual analog scales (an intensity scale and a thermal scale) for each PUNS stimulus where 0 represented the absence of a sensation, 4 indicated the initial discomfort threshold, and 10 represented an unbearable sensation.

### Electroencephalography

Electroencephalographic (EEG) data were acquired using a DC amplifier (BrainAmp MR Plus, Brain Products GmbH, Gilching, Germany) with an actiCap 64 electrode cap (Brain Products GmbH). Data were sampled at 1000 Hz and filtered at DC-250 Hz. All electrodes were prepared with SuperVisc gel (Brain Products GmbH) and electrode impedances were verified <5 kΩ prior to recording.

Participants were seated in a semi-reclined position and provided elbow and forearm support allowing the wrist to be slightly flexed over the front of the armrest. The US transducer was positioned so participants' distal interphalangeal joint rested on the edge of the collar of the transducer with the index finger pad resting on a reservoir of US coupling gel as described above (Figure 1A). Before testing began, for both the PUNS-M and PUNS-T waveforms, participants were briefly acquainted with the stimuli, asked to verbally identify and describe what they felt in response to five randomly spaced stimulus events. During testing, PUNS waveforms were delivered to the volar surface of the index finger every twelve seconds with a positive randomization having of maximum of plus two seconds. Stimuli were delivered in alternating 50 event blocks to the right and left index fingers and counterbalanced across participants. Three blocks were delivered to each finger for a total of 150 stimulations per finger. Testing lasted approximately 1 hour and 15 minutes. Participants were instructed not to look at their finger and asked to fixate on a point on the wall during testing.

### Conventional vibrotactile stimulation

Positioning of participants was similar to that for PUNS during EEG as mentioned above. Participants rested the pad of their index finger upon a piece of 2.5×2.5 cm Velcro™ attached to the dust-cap of a 10 W, 8 Ohm, 10 cm GF100 4X Taiwan speaker. Vibrotactile stimulation was controlled by digitally generating waveforms using a function generator (BK Precision 4078 Function Generator). A 70 Hz square-wave was converted to an analog signal and amplified using an audio amplifier. Stimulus amplitudes were set at approximately twice an individual participant's detection threshold and subjectively reported to be easily detected. All subjects were outfitted with earplugs to dampen any auditory stimulation. A total of 150 stimuli were delivered to the right index finger in three blocks of 50 events. Stimulation was delivered at an inter-stimulus interval of 12 seconds with a positive 2 second randomization.

### Functional magnetic resonance imaging

During functional magnetic resonance imaging (fMRI), the US transducer was affixed to a custom made MR-compatible hand mold that allowed participants to comfortably place their index finger upon the transducer as described above. Prior to scanning, test stimuli were delivered to confirm detectability as described above. Stimuli were delivered every sixth TR (every 12 seconds). A total of 75 stimuli were delivered to each finger in separate runs. Each run lasted approximately 15 minutes.

Functional and anatomical images were collected at Virginia Tech Carilion Research Institute on a Siemens 3T MRI TrioTim scanner using a 12 channel head matrix coil. Prior to functional scans, a T1-weighted magnetization-prepared rapid acquisition gradient echo sequence (MPRAGE) high-resolution anatomical scan (TR  = 2600 msec, TE  = 3.02 msec, flip angle θ = 8°, FOV  = 256×256 mm, slices  = 176, slice thickness  = 1.0 mm) was acquired to align with each subject's BOLD contrast data. Functional images of BOLD contrast signals were acquired using gradient echo echo-planar imaging sequence (TR  = 2000 ms, TE  = 30 ms, flip angle θ = 90°, FOV  = 190 mm, slices  = 33, slice thickness  = 3 mm). Total scan time for each subject was approximately 45 min.

### Data analysis

#### fMRI

Raw data were reconstructed offline and a time series of 450 images was generated for each functional scan (right and left index finger stimulation). The first six volumes which included one stimulus event were discarded from analysis to allow for equilibrium of the magnetic field. The resulting time courses were analyzed using Statistical Parametric Mapping (SPM8; http://www.fil.ion.ucl.ac.uk/spm/). Prior to co-registration, the functional data was slice-time corrected, realigned, preprocessed by linear trend removal, temporal high-pass filtered (128 sec) and three-dimensional motion corrected using a trilinear interpolation. Functional data sets were transformed into MNI space and co-registered with anatomical data for each subject. The resulting time courses were filtered using an 8-mm Gaussian kernel at full width half-maximum. Statistical analysis was performed by fitting the signal time course of each voxel using the general linear model (GLM). The onset of stimulation was used as regressor and modeled using the time derivative of the canonical hemodynamic response function. Additional regressors included head motion parameters generated during fMRI preprocessing.

Individual analysis was performed for Right finger condition and Left finger condition using one-sample t-tests. Testing for significance at the group level (N = 5) was conducted for condition Right finger and condition Left finger using individual one-sample t-tests. Data is presented at *p*<0.001 uncorrected with a cluster threshold of 9 voxels. Areas of activation were identified using the Anatomy probability atlas v1.8 for SPM [21].

#### EEG

Post-processing of EEG data for both vibrotactile SEPs and PUNS USEPs was performed using BrainAnalyzer 2 (BrainProducts GmbH) and EEGLAB [22]. Data were inspected for artifact using a rejection criterion of 100 µV/msec and absolute difference of 150 µV for all channels. Contaminated epochs were eliminated from further analyses. Data was band-pass filtered (1–30 Hz), DC de-trended and re-referenced to the average reference. Data were then segmented into epochs (−200 to 1000 msec), zeroed at stimulus onset, baseline corrected (−200 to 0 msec) and averaged across 100 randomly selected trials. Latency was determined from the peak amplitude of a given potential. Amplitude was determined for a potential as the difference between peak amplitude and pre-stimulus baseline (0 µV). Data are presented as mean ± standard deviation (SD). As is common in studies examining CHEPs, LEPs, and SEPs, the primary electrodes sites we were interested in were CZ, C3, and C4 to assess latency and lateralization. Current source density (CSD) topographic maps were used to evaluate current sources and sinks on the scalp since they can provide a more spatially precise account of cerebral activity compared to the raw voltage distribution due to its lower sensitivity to volume conduction [23] and tissue distortion [24]. CSD maps were generated using the spherical spline interpolation method [25].

## Results

### Subjective ratings of sensations evoked by peripheral ultrasonic neurostimulation

Ultrasound (US) can produce both mechanical and thermal bioeffects on tissues [26], [27] depending on several factors including the acoustic frequency of US used, the duty cycle of waveforms (pulsed versus continuous wave), the types of transducers used (focused versus unfocused), the peak and temporal-average intensities of US waveforms, the total energy delivered, and different US absorption coefficients for tissues [26], [27]. Low-intensity waveforms having a low duty cycle delivered as brief pulses of US for short durations tend to predominantly elicit mechanical effects, whereas high-intensity or high duty cycle waveforms having longer pulse durations (or continuous waves) can lead to tissue heating and thereby elicit both mechanical and thermal effects on tissues. [11], [28].

In the present study, we delivered two distinct pulsed US waveforms to the volar surface of the index finger to achieve pulsed ultrasonic neurostimulation (PUNS) as described in the *Materials and Methods* section. One waveform designated as PUNS-M was designed to elicit a mild vibration sensation, while the other waveform designated PUNS-T was designed to warm the skin while simultaneously producing a vibration sensation. Subjects were asked to confirm the presence of sensations evoked by US by verbally describing them, as well as to rate them using two different visual analog scales (VAS) for each stimulus. One VAS assessed the intensity of a stimulus, while the other VAS assessed the thermal sensation generated by a stimulus. Each scale ranged from 0 representing no sensation (with 4 being the onset of discomfort) to 10 representing an unbearable sensation. Subjects (N = 20) rated the sensation evoked by the PUNS-M waveform as a 2.30±1.45 on the intensity scale and 1.05±0.83 on the thermal scale. The subjects rated the sensation elicited by the PUNS-T waveform as a 3.40±1.50 on the noxious scale and 3.55±1.61 on the thermal scale.

### Ultrasound-evoked potentials triggered by fingertip stimulation with PUNS-M

**Figure 2:**
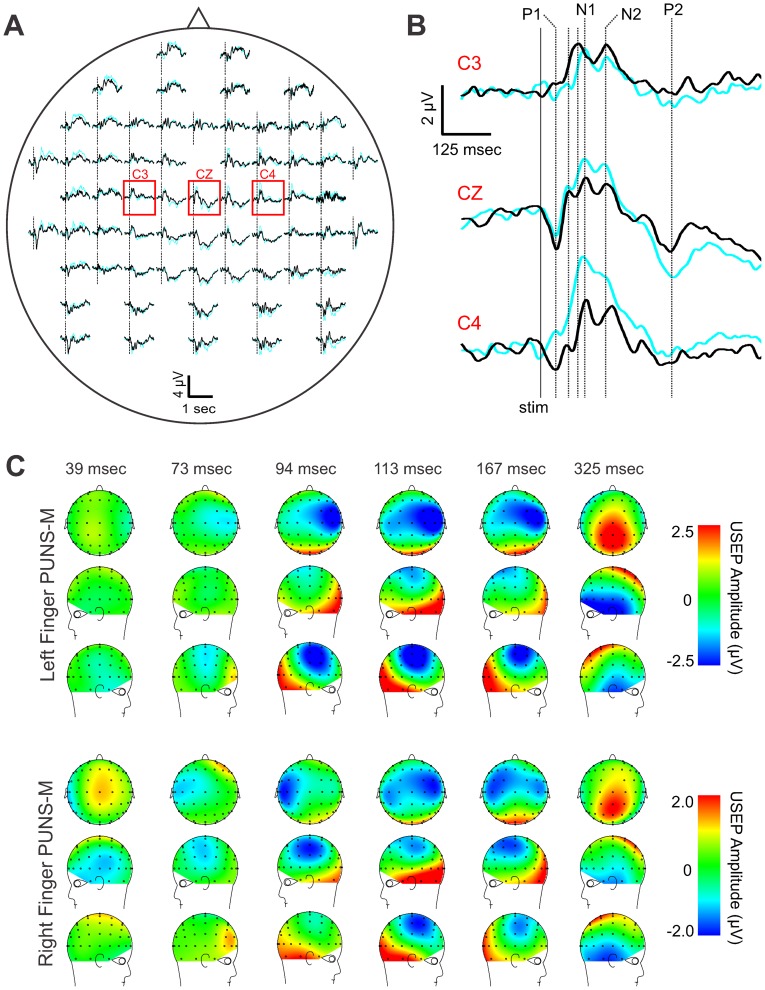
Pulsed ultrasonic neurostimulation elicits ultrasound-evoked potentials. (**A**) Grand average of ultrasound-evoked potentials (USEPs) recorded from five subjects using a 64 channel EEG in response to right (*black traces*) and left (*cyan traces*) fingertip stimulation using a PUNS-M waveform are shown in a top-view of the whole-head. The channels indicated by the *red square* are shown at a higher gain in **B**. The *vertical dashed lines* represent the onset of stimulation and the EEG traces illustrate a time period from 100 msec pre-stimulus to 1000 msec post-stimulus. (**B**) Grand average USEPs from electrode sites C3, CZ, and C4 shown in **A**, but at a higher amplitude and temporal gain. The *vertical dotted lines* show time points of interest for which average voltage maps are shown in **C** and labels (P1, N1, N2, and P2) indicate potentials of interest. (**C**) Topographic voltage maps from time-points corresponding to *vertical dotted lines* in **B** illustrate grand average USEPs obtained in response to PUNS-M stimulation of the left (*top*) and right (*bottom*) fingers.

Delivery of the PUNS-M waveform to the volar surface of the index finger produced quantifiable ultrasound-evoked potentials (USEP) in all participants from which EEG responses were recorded (N = 5). We identified four distinct potentials and named them based on their polarity and temporal order of appearance (P1, N1, N2 and P2). The earliest identifiable potential post-stimulation was a small positive potential (P1). The P1 was clearly evident in electrode site CZ for both right and left finger stimulation and in ipsilateral electrode sites (C3 left index finger stimulation and C4 for right finger stimulation) in all participants (Figure 2A, B). The mean P1 latency at CZ for right finger stimulation was 44.0±1.4 msec and 40.0±5.1 msec for left finger stimulation. Compared to the P1 latencies observed at CZ, the latencies at ipsilateral sites were slightly earlier for both the right (C4 = 40.5±2.1 msec) and left (C3 = 36.5±3.8 msec) finger stimulation. The P1 could not be reliably quantified from contralateral electrode sites.

**Figure 3:**
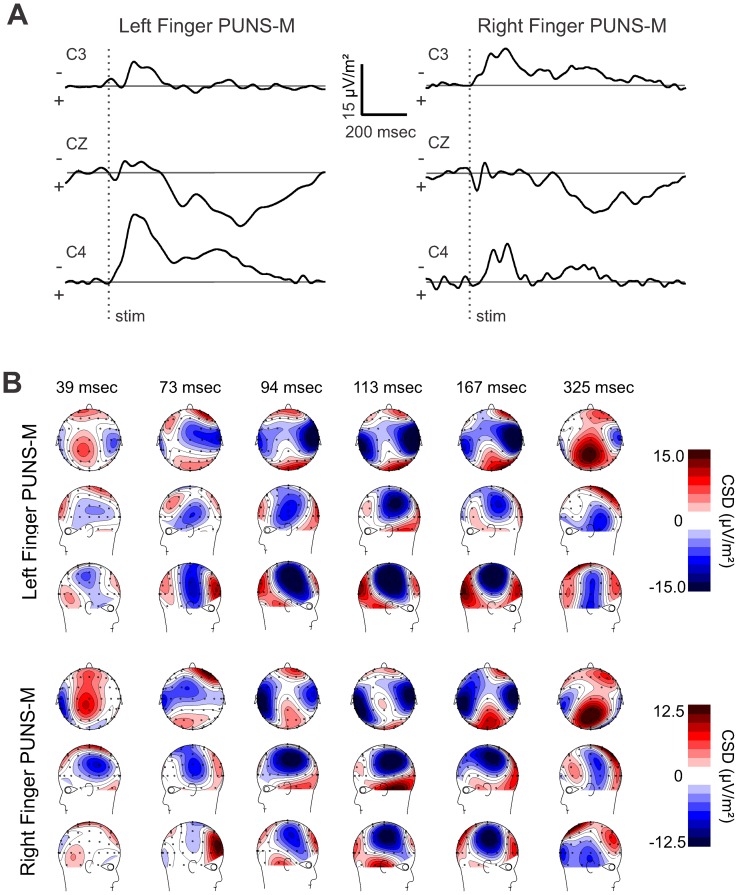
Stimulation of fingertips with PUNS-M reveals dynamic current sources. (**A**) Grand average current source density (CSD) traces obtained from five subjects in response to left (*left*) and right (*right*) fingertip stimulation with PUNS-M waveforms are displayed across time for C3 (*top*), CZ (*middle*), and C4 (*bottom*) electrode sites where (−) indicates current sink and (+) indicates current source. (**B**) Pseudocolor topographic CSD maps corresponding to data illustrated in **A** for left finger (*top panel*) and right finger (*bottom panel*) stimulation with PUNS-M are illustrated for times following stimulation as indicated.

The mean P1 USEP amplitude at CZ was 1.96±1.02 µV for right finger and 1.49±1.32 µV for left finger stimulation with PUNS-M. The mean P1 USEP amplitude at C4 for right finger stimulation was 69% (1.36±0.65 µV) of the CZ amplitude and 53% (0.80±0.41 µV) of the CZ electrode at C3 for left finger stimulation with PUNS-M (Figure 2A, C and Table 1). The CSD maps show a clear contralateral current sink that is maximal underlying parietal electrode sites C4, CP4, C6 and CP6 in response to left finger stimulation with PUNS-M. Similar contralateral sinks were observed at C5 and CP5 in response to right finger stimulation with PUNS-M. These sinks were maximal at slightly more lateral and posterior electrodes (Figure 3B). Both right and left stimulation with PUNS-M also resulted in a central-parietal current source, which was maximal at electrode sites CZ, CPZ, CP1 and C1 (Figure 3B).

**Table 1:** Summary of evoked potential data obtained in response to right fingertip stimulation with pulsed US and vibrotactile sources.

	*Electrode Site*					
	*C3*	*CZ*	*C4*	*C3*	*CZ*	*C4*
**PUNS-M**	**Latency (msec)**			**Amplitude (µV)**		
P1 mean	N/A	44.0	40.5	N/A	1.96	1.36
SD	N/A	1.41	2.08	N/A	1.02	0.65
N1 mean	113.0	117.8	115.0	−2.74	−2.76	−3.24
SD	6.90	6.94	3.26	1.81	1.45	1.45
N2 mean	167.5	167.8	166.3	−2.84	−2.25	−2.57
SD	2.38	5.86	10.80	2.01	2.30	1.55
P2 mean	295.0	304.3	308.5	1.10	1.83	1.45
SD	29.87	35.70	14.71	1.60	1.74	1.21
**PUNS-T**	**Latency (msec)**			**Amplitude (µV)**		
P1 mean	72.0	74.0	68.0	1.04	0.90	0.84
SD	12.73	12.71	14.14	0.82	0.12	1.27
N1 mean	240.5	230.0	228.5	−2.81	−3.67	−3.28
SD	13.40	8.50	10.60	0.84	0.95	0.23
P2 mean	400.5	407.0	405.0	2.32	2.09	2.59
SD	13.44	29.70	31.10	0.48	0.15	0.65
**Vibrotactile**	**Latency (msec)**			**Amplitude (µV)**		
P1 mean	38.0	39.5	N/A	1.58	1.21	N/A
SD	7.10	4.90	N/A	0.12	0.17	N/A
N1 mean	121.5	105	112	−3.25	−3.21	−1.63
SD	10.60	8.50	5.70	2.30	3.40	0.80
P2 mean	187.5	189.5	197.5	1.75	3.91	1.72
SD	2.12	7.77	10.61	1.09	1.65	1.94
N2 mean	336.7	340.0	342.9	−1.34	−1.77	−1.65
SD	10.80	11.60	15.07	1.12	2.03	1.33

In all participants we observed a prominent N1 USEP occurring at multiple electrodes in response to both right and left finger stimulation with PUNS-M waveforms (Figure 2A, C). The N1 latency at CZ for right finger stimulation was 117.8±6.9 msec and 112.3±8.6 msec for left finger stimulation (Figure 2B). The latency of the contralateral N1 was similar for right finger stimulation (113.0±6.9 msec) and left finger (110.8±3.9 msec) stimulation. The N1 latency at ipsilateral electrode sites was similar to that observed at CZ for both right (115.0±3.3 msec) and left finger (117.0±8.0 msec) stimulation. Peak N1 amplitudes were observed at central electrode sites and tended to be largest for both right and left finger stimulation at right-side central parietal electrode sites (Figure 2A, C). The amplitude of the N1 USEP in response to PUNS-M at electrode site CZ measured −2.76±1.45 µV for right finger and −3.21±2.23 µV for left finger stimulation. The mean N1 USEP amplitudes at contralateral electrode sites were −2.74±1.81 µV for right finger stimulation and −4.08±1.85 µV for left finger stimulation using PUNS-M. Conversely the mean N1 USEP amplitudes at ipsilateral electrode sites were −3.24±1.45 µV for right finger stimulation and −2.82±1.73 µV for left finger stimulation with PUNS-M.

The CSD maps revealed a clear progression of a contralateral current sink underlying lateral parietal electrode sites while forming a bilateral current sink under lateral parietal electrode sites corresponding to the time of peak N1 USEP voltages (Figure 3B). For both right and left finger, the current sinks were maximal under electrode site C5, CP5, C6 and CP6 (Figure 3B). The CSD maps also illustrate a robust phase reversal of the N1 from parietal to temporal electrode sites. We further observed a prominent frontal current source appearing prior to the N1 USEP with an additional temporal occipital current source occurring at the peak latency of the N1 USEP (Figure 3B).

Stimulation of index fingers with PUNS-M produced a N2 USEP, which peaked approximately 50 msec after the N1 and was clearly identifiable in central, as well as ipsilateral electrode sites (Figure 2A, B). The mean N2 USEP latencies observed at CZ were comparable for PUNS-M stimulation of right (167.8±5.9 msec) and left fingers (165.5±11.6 msec). Similar mean N2 USEP latencies were observed at both C3 and C4 electrode sites for right (C3 = 167.5±2.4 msec, C4 = 166.3±10.8 msec) and left finger stimulation (C3 = 169.2±12.3 msec, C4 = 158.8±9.2 msec). The mean N2 USEP amplitudes at CZ were −2.25±2.3 µV for right finger and −4.43±2.4 µV for left finger stimulation with the PUNS-M waveform. The mean N2 USEP amplitudes were larger at contralateral electrode sites for both right and left finger stimulation. The mean N2 USEP amplitudes observed for right finger stimulation were −2.84±2.01 µV and −2.57±1.55 µV at C3 and C4 electrode sites respectively (Figure 2 and Table 1). For left finger stimulation the mean N2 USEP amplitudes were −3.89±2.1 µV and −5.62±3.5 µV at C3 and C4 electrode sites respectively (Figure 2 and Table 1). Examination of the voltage maps indicated a similar distribution for N1 and N2 USEPs although the N2 tended to be more confined to the contralateral hemisphere compared to N1 (Figure 2C). A similar distribution was observed through examination of the CSD maps for left finger stimulation where the current sink maximum was isolated to contralateral electrode sites C4, C6 and FC6 while CSD maps produced by right finger stimulation showed current sinks underlying C6, FC6, C5 and CP5 (Figure 3B).

We identified a P2 USEP as the positivity following the descending slope of the N2 USEP (Figure 2B). This P2 was identifiable in all participants although it exhibited highly variable latencies across participants by appearing across a time range spanning from 64 to 138 msec following the peak amplitude of N2 USEPs. The P2 was clearly visible at electrode site CZ and more posterior central sites (Figure 2A, B) although it was not prominent at lateral electrode sites. The mean P2 USEP latency observed at CZ was 304.3±35.7 msec for right finger stimulation and 325.3±27.4 msec for left finger stimulation with PUNS-M. The mean P2 USEP amplitudes observed at CZ were 1.83±1.7 µV for right finger and 2.63±0.9 µV for left finger stimulation (Figure 2B, C and Table 1).

### BOLD responses elicited by fingertip stimulation with PUNS-M

**Figure 4:**
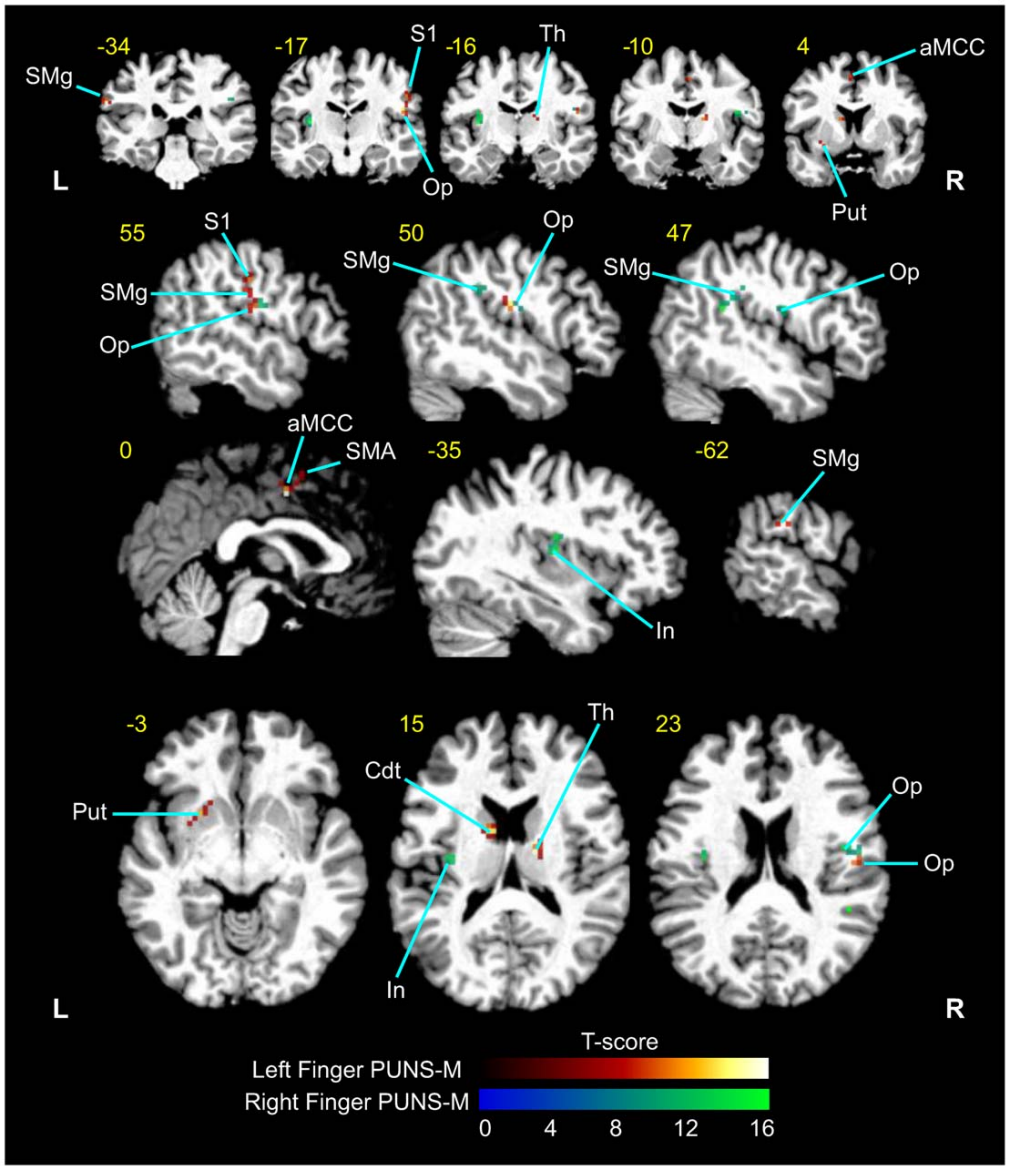
PUNS-M stimulates BOLD contrast signals in somatosensory detection and discrimination networks. Grand average fMRI BOLD responses obtained from five subjects in response to left finger (*red-yellow* LUT) and right finger (*blue*-*green* LUT) stimulation with PUNS-M waveforms (L  =  Left, R  =  right). Anatomical areas shown were significantly activated (p<0.001). The *yellow* numbers correspond to MNI slices in respective views while *white* labels indicate anatomical regions abbreviated as follows: SMg  =  supramarginal gyrus; S1  =  primary somatosensory cortex; Op  =  parietal operculum; Th  =  thalamus; aMCC  =  anterior middle cingulate cortex; Put  =  putamen; SMA  =  supplementary motor area; In  =  Insula; Cdt  =  Caudate.

In order to further explore the effects of peripherally administered US on brain activity patterns we conducted fMRI of BOLD contrast signals in subjects (N = 5) during fingertip stimulation with PUNS-M waveforms. We found that fingertip stimulation with PUNS-M elicited prominent BOLD responses in somatosensory detection, discrimination, and attention networks (*p*<0.001, extent threshold >9 voxels; Figure 4 and Table 2). The areas activated in response to PUNS-M included inferior parietal cortex, supramarginal gyrus, opercular/insular cortex, thalamus, anterior middle cingulate cortex, supplementary motor area, putamen, caudate, and the post-central gyrus (Figure 4 and Table 2).

**Table 2:** Summary of grouped BOLD contrast signals obtained in response to PUNS-M.

X	Y	Z	Anatomy	Cluster (mm^3^)	T-statistic
16	−14	19	Thalamus	162	16.75
15	−7	16	Caudate	324	16.75
51	−16	22	Rolandic Operculum OP3	459	14.24
54	−21	41	S1	162	11.37
54	−16	34	IPC/OP3/OP4	270	11.37
0	−4	43	Middle Cingulate Cortex	594	21.55
−3	5	53	Supplementary Motor Area	486	9.70
−18	14	−5	Putamen	495	17.47
−63	−31	28	Supramarginal gyrus	243	16.62

### USEPs triggered by stimulation of the finger with PUNS-T

**Figure 5:**
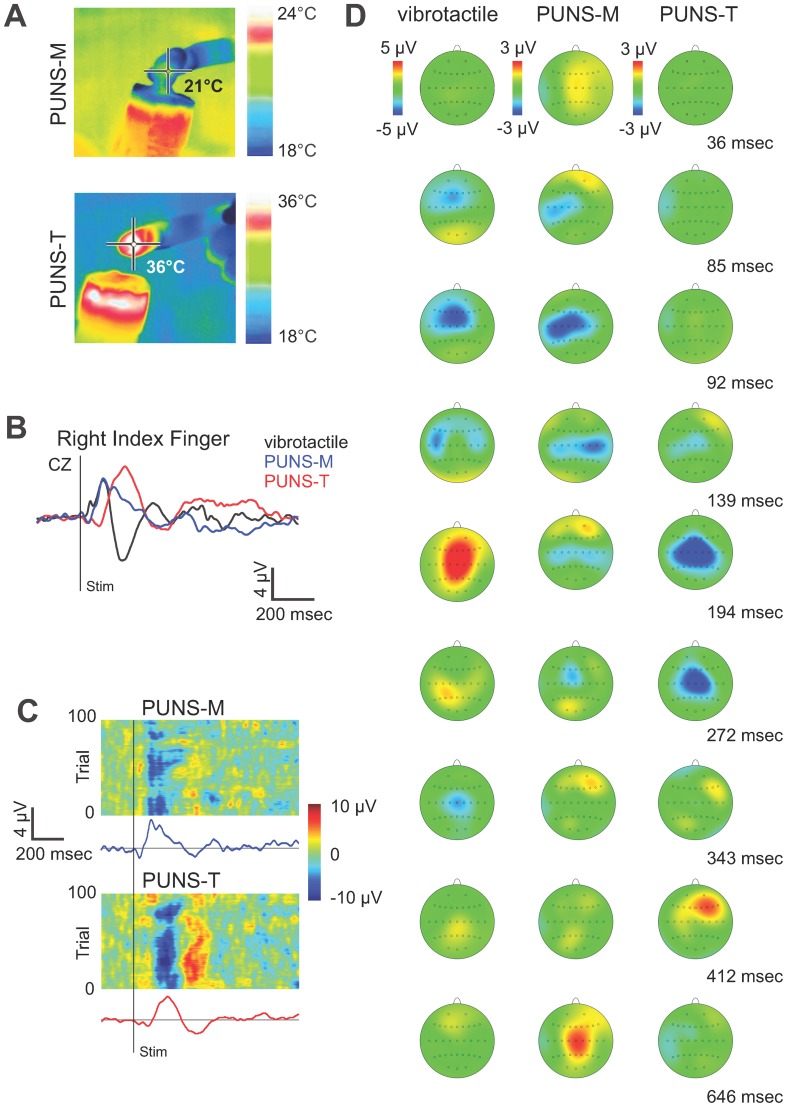
PUNS waveforms can differentially stimulate somatosensory circuits. (**A**) Pseudocolor thermographic images taken with a calibrated infrared camera illustrate the temperature of one participants' index finger immediately following stimulation with a PUNS-M and PUNS-T waveform. (**B**) EEG traces illustrate averaged evoked potentials traces (N = 100) recorded from electrode site CZ from one participant in response to right index finger stimulation with conventional vibrotactile (*black trace*), PUNS-M (*blue trace*), and PUNS-T (*red trace*) waveforms. (**C**) Individual trial responses (N = 100) to PUNS-M (*top*) and PUNS-T (*bottom*) stimulation of the right index finger are shown in the pseudocolor raster plot with corresponding averaged EEG traces below each raster plot. (**D**) Average topographic voltage maps obtained across 100 trials are shown for one participant in response to vibrotactile (*left*), PUNS-M (*middle*), and PUNS-T (*right*) stimulation of the right fingertip. Time points for each pseudocolor voltage map obtained following stimulus onset are indicated.

We next questioned whether PUNS waveforms designed to elicit a mild thermal sensation (PUNS-T) could produce USEPs similar to those observed in response to stimulation with PUNS-M waveforms. Here, we focused on stimulating the right index fingers of subjects with PUNS-T waveforms during simultaneous EEG recordings. We made the primary observations that the PUNS-T waveform used was capable of generating mild heat in the subjects' fingertips (Figure 5A) and elicited robust USEPs (Figure 5B, C). We found PUNS-T stimulation triggered USEPs having a small positivity (P1) followed by a large negativity (N1) and subsequent positivity (P2) observable at multiple electrode sites while being the most robust at central electrode sites (Figure 5B–D).

The mean latencies of these P1 USEPs were 74.0±12.7 msec at the central CZ site, with no clear differences between lateral electrode sites C3 (72.0±12.7 msec) and C4 (68.0±14.1 msec). The corresponding mean P1 USEP amplitudes were 0.9±0.07 µV, 1.04±0.8 µV, and 0.84±1.3 µV at the CZ, C3, and C4 electrode sites respectively. The USEP N1 elicited by PUNS-T stimulation was a large, broad negative potential identifiable from multiple electrode sites having the most prominence at central sites (Figure 5D). Interestingly, the N1 USEP produced by PUNS-T stimulation appears considerably later than the N1 and N2 observed in response to PUNS-M stimulation (Figure 5B, C and Table 1). The mean latencies of N1 USEP triggered by right finger stimulation with the PUNS-T waveform were 230.0±8.5 msec at CZ, 240.5±13.4 msec at C3, 228.5±10.6 msec at C4. The mean amplitudes of N1 USEP elicited by PUNS-T applied to the right index finger were −3.67±0.9 µV, −2.81±0.8 µV, and −3.28±0.2 µV for CZ, C3, and C4 sites respectively (Table 1). The P2 was a large broad positive potential evident following the N1. The latency of the P2 measured 407.0±29.7 msec at electrode site CZ, 400.5±13.4 msec at site C3, and 405.0±31.1 msec at site C4. The mean amplitudes were 2.09±0.15 µV, 2.32±0.5 µV and 2.59±0.7 µV for CZ, C3 and C4 sites respectively (Figure 5B, D).

### BOLD responses elicited by fingertip stimulation with PUNS-T

**Figure 6:**
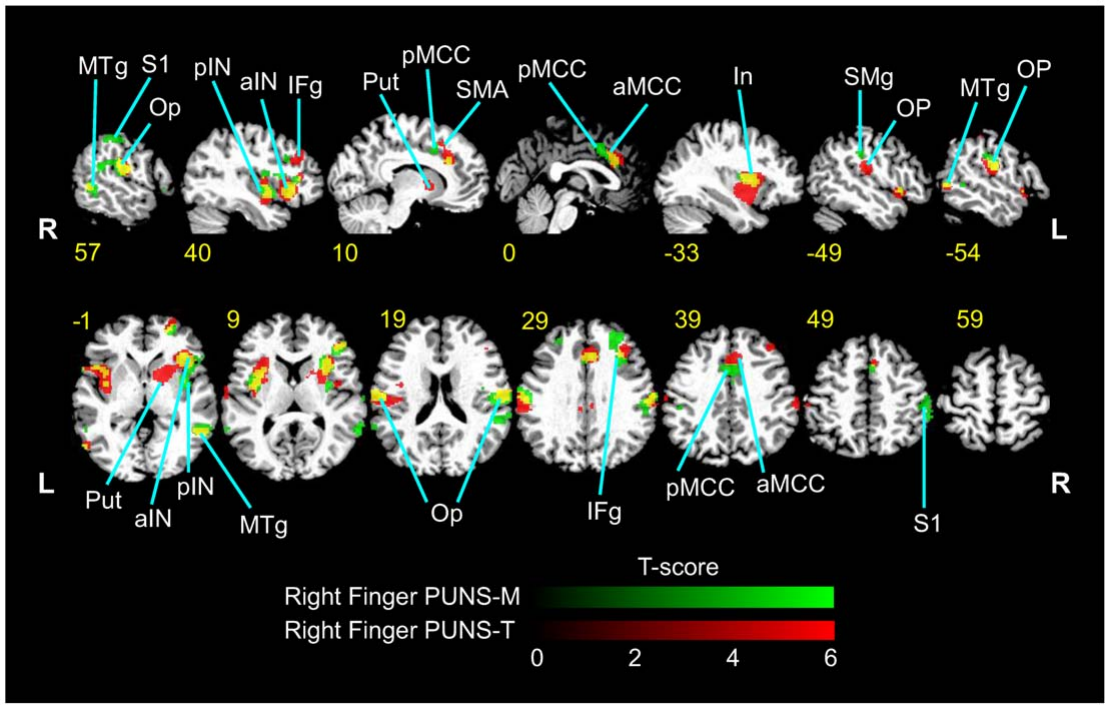
Differential stimulation of somatosensory brain circuits elicited by PUNS as indicated by BOLD contrast signals. Psuedocolor fMRI images illustrating BOLD contrast signals (p<0.001; threshold 9 voxels) are shown for responses to PUNS-M (*green* LUT) and PUNS-T (*red* LUT) stimulation of the right index finger. Areas which were co-activated by both PUNS-M and PUNS-T are indicated by *yellow* voxels. Slice numbers shown in *yellow* text are based on MNI conventions. Anatomical areas activated are indicated by the following abbreviations: MTg  =  medial temporal gyrus; S1  =  primary somatosensory cortex; Op  =  parietal operculum; pIN  =  posterior insula; aIN  =  anterior insula; IFg  =  inferior frontal gyrus; Put  =  putamen; pMCC  =  posterior middle cingulate cortex; SMA  =  supplementary motor area; aMCC  =  anterior middle cingulate cortex; SMg  =  supramarginal gyrus.

Similar to observed during PUNS-M stimulation, we conducted fMRI of subjects undergoing mild thermal stimulation of the right index finger using the PUNS-T waveform. We found that right index finger stimulation with PUNS-T resulted in BOLD responses in multiple brain regions including bilateral rolandic operculum, bilateral anterior and posterior insula, right inferior frontal gyrus, right middle cingulate cortex, right supplementary motor area, right supramarginal gyrus, right middle temporal gyrus, right superior orbital gyrus, right posterior cingulate gyrus, left middle temporal gyrus and left putamen (Figure 6).

### Somatosensory-evoked potentials produced by conventional vibrotactile stimulation of the fingertip

For contrasting purposes we acquired somatosensory-evoked potentials (SEPs) in response to conventional vibrotactile stimulation of particpants' right index finger. Vibrotactile stimulation resulted in an early small positivity (P1) and a large negative/positive/negative (N1/P2/N2) complex. The mean SEP P1 latencies were 39.5±4.9 msec at CZ (Figure 5B) and 38.0±7.1 msec at the contralateral C3 site. The SEP P1 was not reliably quantifiable at the ipsilateral C4 site. At the CZ site, the latency of the SEP N1 produced by vibrotactile stimulation was 105.0±8.5 msec while the P2 was 189.5±7.8 msec and the N2 was 340±11.6 msec. At site CZ, amplitudes were 1.21±0.2 µV, −3.21±3.4 µV, 3.91±1.65 µV and 1.77±2.03 µV for the P1, N1, P2 and N2 respectively. In general, the SEPs we observed in response to vibrotactile stimulation displayed sharper and more clear inflections compared to the USEPs produced by PUNS-M. In addition, there was a polarity reversal of USEPs elicited by PUNS-T compared to the SEPs triggered by vibrotactile stimuli (Figure 5B). Chronological comparison of the topographic voltage distributions for vibrotactile, PUNS-M, and PUNS-T is displayed in Figure 5D.

## Discussion

We utilized two distinct ultrasound (US) waveforms as PUNS stimuli applied to the index fingers of subjects. The waveform evoking a mechanical sensation (PUNS-M) was reported by subjects to elicit a unique, non-noxious buzzing feeling, while the other waveform (PUNS-T) produced a thermomechanical sensation described by subjects as a warm buzzing sensation. These PUNS waveforms resulted in distinct USEP morphologies and BOLD activation patterns encompassing previously described somatosensory detection and discrimination circuits shown responsive to more conventional stimulation devices eliciting mechanical, thermal, and painful sensations.

### Ultrasound-evoked potentials

The USEP occurring in response to fingertip stimulation with PUNS-M had a morphology primarily defined by an early, central-parietal positivity (P1) and a larger double-peaked central negativity (N1 and N2; Figure 2B). This USEP morphology shares some similarities to prior descriptions of EPs elicited using other sensory stimulation modalities, such as LEPs [29], CHEPs [30], and SEPs elicited by electrical stimulation [31]. The latency of the P1 is consistent with Aβ fiber and primary somatosensory (S1) activation [31]. Potentials in this time range are not evident from laser or contact heat stimulation [29], [30]. The N1 USEP generated by PUNS-M exhibited a latency (Figure 2B) and topography (Figures 2C and 5D) similar to SEPs previously recorded from secondary somatosensory cortex (S2)/parietal operculum (Op) [31], [32]. Innocuous median nerve stimulation of primarily Aβ fibers using electrical pulses produces S2/Op SEPs, which have latencies ranging from 60 msec [33] to 120 msec [32]. Considering the P1 and N1 USEP latencies (Table 1) combined with the observations that PUNS-M stimuli did not elicit skin heating (Figure 5A) or induce discomforting sensations, it seems likely PUNS-M waveforms stimulated low-threshold Aβ fibers. Further supporting this contention, the P1 and N1 USEP exhibited latencies nearly identical to SEPs obtained in response to vibrotactile stimulation (Figure 5B), which is known to involve the activation of low-threshold Aβ fibers [3], [4]. From our observations alone however, we cannot rule out the stimulation of other fibers by PUNS-M waveforms since the N2 USEP latencies we observed are in good agreement with previous reports of Aδ activation [34], [35].

As mentioned above PUNS-M stimuli activated somatosensory circuits through a mechanical (non-thermal) mode of action as indicated by the absence of tissue heating (Figure 5A). Ultrasound is well known to be capable of inducing both mechanical and thermal bioeffects on tissues [26], [27]. Further, previous studies have reported US can stimulate mechanical and thermal sensations in the human hand [11], [28]. As such, we naturally questioned how PUNS waveforms acting partially through thermal mechanisms would differently affect somatosensory circuit responses. It has been previously reported that warm sensations (30–32°C) result in the appearance of late onset SEPs (appearing ≈ 470 msec post-stimulus) due to the activation of slow conduction velocity (2.5 m/sec) C-fibers [34]. In response to PUNS-T, which maximally heated the fingertip to 36°C (Figure 5A), we observed a P2 USEP having an onset latency of about 407 msec at CZ. The kinetics of this P2 USEP indicate PUNS-T may have partially stimulated C-fibers in a manner similar to previous observations of C-fiber activation in response to skin warming [34].

The USEPs triggered by PUNS-T were kinetically different from CHEPs previously described in response to Aδ fiber activation with noxious heat [6], [7]. These differences are likely due to several factors, such as the rates at which the skin was heated or the maximum skin temperatures generated by PUNS-T compared to peltier devices. Additionally the polymodal nature of PUNS-T (both mechanical and thermal) likely led to the stimulation of a broader population of receptors and fibers compared to surface heating alone as observed for CHEPs. The ≈ 230 msec latency of the N1 USEP we observed in response to PUNS-T are slower than would be expected for Aβ activation, but are consistent with Aδ fiber stimulation achieved using lasers [34], [35], [36]. Besides heat, Aδ afferents respond to both damaging and non-damaging pressure [3], [4]. Thus, we hypothesize PUNS-T preferentially activated a population of thermosensitive C-fibers and polymodal Aδ fibers compared to low-threshold Aβ fibers, which are more robustly activated by PUNS-M. More detailed neurophysiological recordings across a wider range of PUNS waveforms will be required before the exact fiber contributions giving rise to USEPs can be further delineated.

### BOLD activation patterns in response to peripheral ultrasonic neurostimulation

Fingertip stimulation with PUNS-M produced BOLD responses of contralateral somatosensory areas in the post-central gyrus and rolandic operculum areas similar to conventional mechanical stimulation [37], [38], [39]. In addition to these somatosensory discrimination areas, several other cortical and sub-cortical areas were activated by PUNS-M. These areas included the thalamus, anterior middle cingulate cortex (aMCC), supplementary motor area (SMA), supramarginal gyrus (SMg), insula and striatum (Table 2). Anterior MCC activity has been attributed to stimulus discrimination [40] and intensity coding [41], [42] whereby its activation occurs below pain thresholds and is thought to provide a cognitive component of stimulus awareness. Such activation of the aMCC appears to be consistent with our observations since PUNS-M was not subjectively reported to elicit discomfort.

It is thought SMA activation may be the result of attention directed to a somatosensory stimulus [43] for evaluation and discrimination purposes, perhaps due to stimulus novelty [44]. The SMg has been shown to be active during hierarchical tactile activity, such as tactile pattern recognition [45] and feature or object discrimination and exploration [44], [46]. Despite no requirement for object exploration or feature discrimination, it is possible the SMA and SMg were activated due to increased awareness to a novel stimulus. This possibility seems likely since participants routinely described the feeling evoked by PUNS-M as “weird buzzing” or a sensation not previously experienced. Taking these observations into consideration, we hypothesize activation of the SMA and SMg in response to PUNS-M is linked to a combination factors including the diversity of receptors and fibers activated, as well as a specific awareness to the stimulus or attention to its novelty. To evaluate whether the SMA and SMg are indeed involved in detecting novel sensations elicited by US, future studies should determine if activity in these networks subside as individuals accumulate PUNS experience and familiarity.

We observed overlapping BOLD signals in response to the different PUNS waveforms, although there was considerably more activation in the anterior insula and anterior cingulate cortex in response to PUNS-T compared to PUNS-M (Figure 6). Previous findings suggest the anterior insula and anterior cingulate cortex are associated with affective responses to painful stimuli [8], [9], [10]. Further, the BOLD signals in response to stimulation with PUNS-T waveforms are similar to those observed in studies using noxious, contact heat and laser stimuli to uncover patterns of brain activity identified as the “pain matrix” [47]. Thus based on both the USEP morphology and BOLD signals we observed, it appears PUNS-T waveforms can activate pain pathways despite the fact that subjective reports indicated these waveforms were not necessarily noxious. Future refinement and investigation of US waveform parameters to elicit isolated sensations of heat, vibration, and pain will allow for a more detailed decomposition of the fibers and pathways underlying particular brain activity patterns evoked by PUNS while increasing our understanding of how US produces various bioeffects. We feel confident in concluding however that pulsed US is capable of differentially activating brain networks involved in both primary sensory perception and tactile discrimination or attention.

### Ultrasound waveform considerations for peripheral neurostimulation

In the present study we used US waveforms (0.35 MHz) having two different acoustic intensity values (PUNS-M = 11.8 W/cm^2^ and PUNS-T  = 54.8 W/cm^2^) to trigger different sensations in the fingertip. As discussed above our EEG data indicate PUNS-M led to the activation of a population of low-threshold Aβ fibers, while PUNS-T led to the activation of a population of Aδ and C-fibers. Previous studies have provided subjective reports that US could differentially stimulate sensations across a range of acoustic frequencies (0.48 to 2.67 MHz) and intensities (8–3000 W/cm^2^; [11], while studies examining EP responses to painful ultrasonic stimulation of joints in humans employed an acoustic intensity of 1125 W/cm^2^ delivered by 0.5 MHz US [14], [15]. The EP latencies reported by Wright and colleagues (1989, 1993) are similar to those obtained with PUNS-T in the present study both of which are consistent with Aδ fiber stimulation achieved using lasers [34], [35], [36]. We postulate however differences in the acoustic intensity and stimulus durations led to activation of different populations of high- and low-threshold fibers. This seems likely since subject in the studies conducted by Wright and colleagues (1989, 1993) reported moderate to intense pain in response to US stimuli, while are participants in our study reported no discomfort or pain in response to either PUNS-M or PUNS-T stimuli.

Using focused ultrasound (1.1 MHz) across a range of acoustic intensity values to stimulate sensations in humans, prior functional observations using two-point discrimination tasks have indicated mechanoreceptor density is a major factor in determining detection thresholds whereby most individuals can feel ultrasonic stimuli at intensity values <100 W/cm^2^ [13]. In our study we found 100% of the subjects tested (N = 20) were capable of detecting PUNS stimuli while Dickey and colleagues (2012) reported about 3% and 10% of the subjects they tested could detect the US stimuli at temporal average intensity values similar to our PUNS-M and PUNS-T waveforms respectively. Variation in detection sensitivity to an US waveform could be due to acoustic frequency differences between the studies. In fact the mechanical index of US (a dimensionless number indicating potential mechanical bioeffects caused by cavitation) rises as the acoustic frequency decreases. Consistently, Gavrilov and colleagues (1976) reported that tactile sensations could be experienced in the fingertip of humans in response to a 0.48 MHz stimulus at acoustic intensity of 8 W/cm^2^, but the intensity had to reach 80 W/cm^2^ for the same sensation to be generated by 1.96 MHz US and 120 W/cm^2^ for 2.67 MHz. Differences in the stimulus durations could also explain differences in detection sensitivities across various US stimuli. We implemented stimuli durations of 0.5 sec for PUNS-M and 1 sec for PUNS-T while Dickey and colleagues (2012) employed a shorter, 0.1 sec stimulus duration. Modulating the total stimulus duration will likely enable different populations of slowly- and rapidly-adapting mechanoreceptors to be recruited by US. Yet another explanation for the differences described above could be that highly focused US transducers used by Dickey and colleagues (2012) may lead to the generation of shear waves at the fringes of acoustic fields in tissues, whereas unfocused or scattered US may be less likely to generate such transverse waves in tissues (see discussion below). More in depth examinations of these issues should be the focus of future investigations employing US for peripheral neurostimulation.

In the present study we implemented pulsed US for peripheral stimulation, whereas all prior studies examining the influence of US on somatosensory circuits employed continuous wave (CW) US. The use of pulsed US can confer several advantages over CW US for neurostimulation. For example, temperature increases are slower to rise and the likelihood of transient or inertial cavitation is reduced when delivering US in a pulsed mode. During pilot studies leading up to the design of the PUNS-M and PUNS-T waveforms used in this study, we found different sensations could be generated by changing duty cycle or pulse repetition frequency and/or pulse duration of US waveforms while keeping the total stimulus duration and acoustic frequency constant (data not shown). As our studies clearly illustrate it is possible to achieve differential peripheral neurostimulation by utilizing the mechanical bioeffects, as well as the thermomechanical effects of US on tissues. Implementing pulsing strategies (versus CW) for the design of ultrasonic neurostimulation waveforms greatly expands the parameter space needing to be examined by future investigations in order to determine how specific US can be for differential neurostimulation.

### Potential mechanisms of action underlying the influence of ultrasound on neural structures

The somatosensory system is known to dynamically respond to a variety of mechanical and thermal stimulus modalities across a broad range of stimulus frequency space [3], [4], [48]. Based on our observations described above, as well as the observations of others [11], [13], [14], [15], [28] it appears US waveforms can be tuned for the stimulation of specific fibers, thermosensitive and mechanosensitive ion channels, or other receptor types. The mechanisms of action underlying how US might achieve such differential neurostimulation need to be more thoroughly explored. The actions of US on biological systems are complex and several critical issues should be examined especially with respect to the mechanical bioeffects of US.

Besides the direct effects of acoustic radiation forces on cells, US can induce cavitation of gas bodies (bubbles) in soft tissues [26], [27]. The probability of cavitation is dependent on several factors including the size of gas bodies residing in tissues, the duty cycle of US waveforms, and the frequency and intensity of US employed [49], [50]. There are two major types of cavitation, which should be considered here. Stable cavitation refers to the oscillation of small gas bodies in acoustic fields that trigger other mechanical effects, such as acoustic streaming. On the other hand, transient (inertial) cavitation refers to the non-linear oscillation and violent collapse of larger gas bodies producing shock waves associated with locally high pressure levels and temperature rises in tissues. Whether or not stable cavitation contributes to the ability of US to stimulate peripheral somatosensory systems is not currently known and will be hard to determine since it is difficult to quantify. While transient cavitation can likely produce effects in soft tissues capable of stimulating peripheral sensory receptors, we argue that it not a mechanism of action underlying the effects of PUNS we have observed here. This is due to the fact that the threshold for transient cavitation in swine muscle has been reported to be 75±8 W/cm^2^ (higher than the intensities we used for PUNS) at an acoustic frequency of 0.386 MHz (similar to the 0.35 MHz we used for PUNS) [51]. Other reports of cavitation thresholds also support our position that transient cavitation is not a likely mechanism of action mediating the PUNS waveforms used in our study [49], [50], [52].

The existence of various wave modes in tissues exposed to acoustic energy may contribute to different mechanisms of action underlying the ability of US to achieve neurostimulation. For example, US may propagate through tissues as a longitudinal wave or a shear (transverse) wave. At present it is not currently known if or how these two different wave modes affect phospholipid membrane conductance, ion channel activity, or sensory receptors. It does appear reasonable however, that differences in shear stresses and tension generated by US in tissues would lead to different actions on membranes and membrane-bound receptors. Numerous ion channels and polymodal receptors like members of the transient receptor potential (TRP) superfamily involved in sensory transduction processes are known to be activated by mechanical pressure, stress, and tension [53], [54], [55]. Determining whether forces exerted by acoustic pressures transmitted using US can alter the activity of these channels and receptors should indeed be the target of future studies.

In addition to channel proteins and receptors, other subcellular components of cells are also mechanically sensitive. Cytoskeletal elements such as actin, microtubules, and intermediate filaments are well known to be capable of transducing and sensing mechanical forces in cells [56], [57], [58]. It has in fact been suggested that US may achieve neuromodulation by acting upon microtubules to influence their electro-mechanical resonance properties occurring in the low MHz range [59]. Whether US influences microtubules or other cytoskeletal proteins to achieve peripheral neurostimulation is not known, but should be explored. There are certainly many mechanosensitive features of cells by which US could act to alter activity. Understanding the mechanisms of action by which US affects cellular activity will be a key to its future use in neurostimulation embodiments. However, it is of critical importance to gain a broader understanding of these actions given that US represents the most widely implemented method of diagnostic imaging used in healthcare today and we still do not fully understand how it affects cellular physiology and homeostasis.

### Conclusions

Pulsed US applied to the finger elicits EPs having characteristics both similar to and unique from those evoked by conventional innocuous and noxious stimulus modalities, such as vibrotactile stimulation, transcutaneous electrical nerve stimulation, contact heat stimulation, and laser stimulation. Pulsed US waveforms for PUNS can be modulated and tailored to differentially stimulate a diverse population of Aβ, Aδ, and C-fibers resulting in the distributed activation of somatosensory brain networks. It has been previously noted that lasers are the best available tool for assessing small fiber function [60]. It seems that statement may need to soon be amended to include US, which has the unique capability of activating a variety of fiber types depending upon the needs of the investigator or clinician. First however, future studies should employ US for somatosensory stimulation to confirm our findings, as well as observations made previously by others [11], [13], [14], [15], [28] while expanding upon them to elucidate the mechanisms of action underlying the mechanical bioeffects of US on neurons and their subcellular components.
